# Drug repositioning can accelerate discovery of pharmacological chaperones

**DOI:** 10.1186/s13023-015-0273-2

**Published:** 2015-05-07

**Authors:** Bruno Hay Mele, Valentina Citro, Giuseppina Andreotti, Maria Vittoria Cubellis

**Affiliations:** Department of Agricultural and Food Sciences, University Federico II, Naples, Italy; Department of Biology, University Federico II, Naples, Italy; Istituto di Chimica Biomolecolare –CNR, Pozzuoli, Italy

**Keywords:** Pharmacological chaperone, Drug repositioning

## Abstract

A promising strategy for the treatment of genetic diseases, pharmacological chaperone therapy, has been proposed recently. It exploits small molecules which can be administered orally, reach difficult tissues such as the brain and have low cost. This strategy has a vast field of application. In order to make drug development as fast as possible, it is important to exploit drug repositioning. We evaluated the impact and limitations of this approach for rare diseases and we provide a shortcut in finding drugs for off-target usage.

**Electronic supplementary material:**

The online version of this article (doi:10.1186/s13023-015-0273-2) contains supplementary material, which is available to authorized users.

## Correspondence/Findings

A large share of mutations associated to human diseases causes the destabilization of specific proteins. The activity of unstable proteins can be rescued by small molecules that act as pharmacological chaperones (PC). Usually PC are inhibitors or antagonists of their targets used at a very low concentration, but other types of molecules such as activators or allosteric ligands, which do not reduce activity, would be more appropriate [1-3]. There is a limit in this approach because not all the genotypes of a given disease are eligible for therapy with PC and only in some cases is it possible to predict the responsiveness of specific mutations [4].

Drug repositioning could accelerate the discovery of PC. The first successful case is provided by imino-sugars that interfere with N-glycosylation in cells infected by enveloped viruses, deoxynojirimcin and its derivatives [5]. Clinical trials for the treatment of HIV with deoxynojirimcines were unsuccessful, because the antiviral concentration required could not be achieved in humans. However the same imino-sugars could be used as PC at low concentration for a different target, glucosylceramidase (Uniprot: P04062), to treat Gaucher disease (MIM: 230800) [6] and lysosomal alpha-glucosidase (Uniprot: P10253), to treat Pompe disease (MIM: 232300) [7,8]. The usage of imino-sugars was then extended to other lysosomal glycosidases to cure some storage disorders.

**Figure 1:**
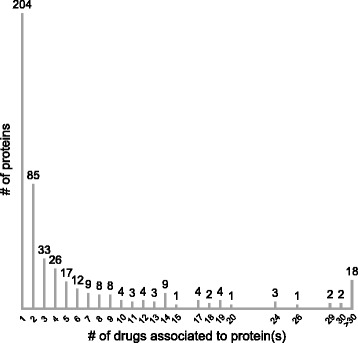
Orphan_Protein distribution per drug. Proteins associated to rare diseases are ordered by a number of interacting small molecule FDA-approved drugs.

Drug repositioning should be run systematically for the discovery of PC. To support our proposal we gathered all the proteins that are associated to rare diseases, i.e. the entries that have a link to Orphanet [9] in Uniprot (Orphan_proteins). For 608 entries out of a total of 3289 Orphan_proteins we found a link to DrugBank, a database including FDA-approved small molecules, experimental and nutraceuticals drugs [10]. DrugBank annotates each record with the known pharmacological protein target, but also with other proteins that are activated or inhibited by the drug. In the vast majority of cases, links between Orphan_proteins and drugs only indicate relations documented in the literature, but do not implicate a recognized pharmacological action of the drug on the target. The histogram in Figure 1 shows that several Orphan_proteins interact with one or more approved small molecules and the list is provided in Additional file 1. Since our aim is to support the usefulness of repositioning, we excluded biotech drugs because some of them have already been approved for enzyme replacement therapy of rare diseases. We also excluded cytochromes, which contribute to the metabolism of many drugs. Small chemicals that interact with Orphan_proteins are excellent starting points to develop PC. A proof of concept is represented by a paper which appeared in 2015 [11] where a screening of 3200 known drugs from commercial compound libraries led to the identification of Ibuprofen as a corrector of the transmembrane conductance regulator (CFTR) (Uniprot: P13569). Ibuprofen has a pharmacological action on Prostaglandin synthase 1 and 2 (Uniprot: P35354, P23219), but besides this action, DrugBank reports the inhibition of CFTR based on a paper published in 1998 [12].

In addition, we found several other cases in which small approved drugs were successfully repositioned as PC for rare diseases: doxorubicin, an anti-neoplastic anthracycline, for Cystic fibrosis (MIM: 219700) [13], Diltiazem, an antihypertensive, for Gaucher disease (MIM: 230800) [14], Ambroxol, a mucolytic agent, for Gaucher and for Fabry disease (MIM: 301500) [15,16], Acetylcysteine**,** another mucolytic agent, for Pompe disease (MIM: 232300) [17], Pyrimethamine, an anti-parasitic compound, for GM2 gangliosidosis (MIM: 272800) [18], carbamazepine, a dibenzazepine, for Hyperinsulinemic Hypoglycemia (MIM: 256450) [19] and Salicylate for Pendred Syndrome (MIM: 274600) [20].

In these cases, however, the link between the drug and the Orphan_protein, could not be found in DrugBank. This absence of annotation shows how difficult it is to mine the literature and we admit that also our list of approved drugs tested as PC may be incomplete. The development of drugs for rare diseases would benefit from a mechanism that favours the deposition of data concerning the interaction of small molecules and proteins into databanks.

## Additional file

Additional file 1:
**Orphan_Proteins associated to approved small molecules are listed.** The column headings are: Entry, Uniprot ID; Entry Name, Uniprot entry name; Status, reviewed if SWISS-Prot entry; Protein names, protein full name; Gene names, Names of each gene associated to the protein; DrugBank cross-reference, List of drugs associated (by DrugBankID); Orphanet cross-reference: List of orphan diseases (by OrphanID) associated to the protein.

## Abbreviations

PC: Pharmacological chaperone
CFTR: Cystic fibrosis transmembrane conductance regulator
